# A qualitative study on barriers in the prevention of anaemia during pregnancy in public health centres: perceptions of Indonesian nurse-midwives

**DOI:** 10.1186/s12884-015-0478-3

**Published:** 2015-02-26

**Authors:** Widyawati Widyawati, Suze Jans, Sutarti Utomo, Jeroen van Dillen, ALM Lagro Janssen

**Affiliations:** School of Nursing, Faculty of Medicine, Universitas Gadjah Mada, Yogyakarta, Indonesia; Department of Primary and Community Care, Gender & Women’s Health, Radboud University Medical Center, 117, P.O. Box 9101, 6500 HB Nijmegen, The Netherlands; Royal Dutch Organisation of Midwives (KNOV), Nijmegen, Netherlands; Provincial Health Office of Yogyakarta Special Province, Yogyakarta, Indonesia; Department of Obstetrics and Gynaecology, Radboud University Medical Center, Nijmegen, The Netherlands

**Keywords:** Nurse-midwives, Competences, Anaemia, Pregnant women, Antenatal care, Comprehensive approach

## Abstract

**Background:**

Anemia in pregnancy remains a major problem in Indonesia over the past decade. Early detection of anaemia in pregnancy is one of the components which is unsuccessfully implemented by nurse-midwives. This study aims to explore nurse-midwives’ experiences in managing pregnant women with anaemia in Public Health Centres.

**Methods:**

We conducted a qualitative study with semi-structured face to face interviews from November 2011 to February 2012 with 23 nurse-midwives in five districts in Yogyakarta Special Province. Data analysis was thematic, using the constant comparison method, making comparison between participants and supported by ATLAS.ti software.

**Results:**

Twelve nurse-midwives included in the interviews had less than or equal to 10 years’ working experience (junior nurse-midwives) and 11 nurse-midwives had more than 10 years’ working experience (senior nurse-midwives) in Public Health Centres. The senior nurse-midwives mostly worked as coordinators in Public Health Centres. Three main themes emerged: 1) the lack of competence and clinical skill; 2) cultural beliefs and low participation of family in antenatal care programme; 3) insufficient facilities and skilled support staff in Public Health Centres. The nurse-midwives realized that they need to improve their communication and clinical skills to manage pregnant women with anaemia. The husband and family involvement in antenatal care was constrained by the strength of cultural beliefs and lack of health information. Moreover, unfavourable work environment of the Public Health Centres made it difficult to apply antenatal care the pregnant womens’ need.

**Conclusions:**

The availability of facilities and skilled staffs in Public Health Centre as well as pregnant women’s husbands or family members contribute to the success of managing anaemia in pregnancy. Nurse-midwives and pregnant women need to be empowered to achieve the optimum result of anaemia management. We recommend a more comprehensive approach in managing pregnant women with anaemia, which synergizes the available resources and empowers nurse-midwives and pregnant women.

## Background

Anaemia in pregnancy remains a major problem in Indonesia [1,2]. Anaemia in pregnancy is defined as a condition where the level of haemoglobin (Hb) in the blood is less than 11 g/dl [3]. Based on data of the Indonesia Health Survey 2007, the prevalence of anaemia in pregnancy in Indonesia is 28% [4], with a similar percentage found in Yogyakarta in 2009 [5]. Studies have suggested that the main cause of anaemia in pregnancy in Indonesia is iron deficiency [1,2,6], resulting from the insufficient consumption of foods containing iron, vitamin A and folic acid, and from the presence of hookworms [2,7,8]. In several places in Indonesia, anaemia is also caused by HIV and malaria [9,10].

Considering the impact of anaemia on the outcome of pregnancy, pregnant women need to receive an adequate antenatal care. Adequate antenatal care prevents the women and the unborn baby to have health problems [11]. In order to provide an adequate antenatal care, in 2007 the Ministry of Health decreed the standard of midwifery care which consists of antenatal care, intranatal care, postnatal care, neonatal care, healthy child care under five, and reproductive period care [12]. Indonesian antenatal care standard consists of 11 procedures that have to be accomplished by nurse-midwives [13]. These procedures are 1) weight measurement, 2) upper arm circumference measurement, 3) blood pressures measurement, 4) fundal height measurement, 5) fetal heart rate measurement, 6) determine fetal presentation, 7) provide tetanus toxoid immunisation, 8) provide iron tablet, 9) provide laboratory test, 10) provide referral properly, 11) provide health education. To implement these procedures correctly, every Public Health Centre is required to develop a technical procedure or technical guideline, which describes in details on how the nurse-midwives should implement these procedures promptly [13].

According to the Indonesia Demographic and Health Survey 2002 and 2012, one time antenatal care visit and four time antenatal care visits had respectively a coverage of 94.9% and 81.0% which is quite high [14,15]. Despite the high percentages of antenatal care visits, it has not represented the quality of antenatal care given. The studies on the quality of antenatal care related to anaemia prevention in Indonesia shown that 30% of pregnant women who attend antenatal care in Public Health Centres have not received iron tablet, and 40% of the pregnant women have not been informed about signs of pregnancy complications [16]. Moreover, only 58% of pregnant women get an early examination of signs and symptoms of anaemia during pregnancy [17].

The background to why these procedures are not carried out is unknown. More insight into the difficulties in implementation of the antenatal care standard to detect anaemia early in pregnancy would support policy maker and nurse-midwives in achieving a clinical practice which is well tailored to the pregnant women’s needs.

Our study aims to explore the experiences of the nurse-midwives in Yogyakarta Special Province on how they carry out antenatal care for pregnant women with anaemia, as well as to provide insight into their perceived competencies in prevention of anaemia.

## Methods

### Design

We used a qualitative method with semi-structured interviews.

### Setting and sample

#### Characteristics of the setting

The data were collected in the main Public Health Centres located in Yogyakarta Special Province, in Indonesia [18]. In total, there are 24 main Public Health Centres with a total of 264 nurse-midwives responsible for the provision of health care services for almost 3.5 million inhabitants. All mother and child health care services in Public Health Centres are conducted by nurse-midwives.

#### Sample and recruitment

The inclusion criteria of participants were: being a nurse-midwife with a formal educational background in nurse-midwifery at diploma level, with at least two years of antenatal work experience, employed by a Public Health Centre and resident of Yogyakarta Special Province. The head of the Public Health Centre chose one of the nurse-midwives as a representative from each public health centre.

#### Data collection

The interview guide was based on the existing literature about implementation of antenatal care services including the standard component of early detection on anaemia in pregnancy [19-21] as well as expert (senior nurse-midwives’) opinion. The topics included: the nurse-midwife’s experience in applying the current antenatal care standard; the nurse-midwife’s perception of her competencies to manage pregnant women with anaemia; the nurse-midwife’s perception of patients’ and their family’s experience of antenatal care; and the availability of supporting resources such as medical facilities and human resources in the Public Health Centres.

At the start of the interview additional information was collected about participants’ characteristics, such as age, years of experience as a nurse-midwife, and details of training followed during the last five years.

All interviews were carried out between November 2011 till February 2012 by two senior nurses experienced in qualitative interviewing. Each interview lasted around 30 minutes. To minimize disturbances to the daily running of the clinics, interviews were scheduled before or after the working hours, located in a private room in the Public Health Centre. After 23 interviews the data collection was stopped because no new themes were emerging and therefore we concluded saturation was achieved. Interviews were fully recorded and anonymously transcribed by the interviewers. An observer used a log book (research diary) to record non verbal aspects of the interviews.

## Data analysis

The process of data analysis was led by the primary researcher (WW). Data analysis was thematic, using the constant comparison method of noting and coding emerging themes, and making comparisons between participants. Each transcript was coded by two members of the research team (WW and SU). Quotes have been selected to illustrate the themes that emerged from the interviews and have been translated into English. ATLAS.ti software package was used to support the analysis of the transcripts.

There are two types of positions for nurse-midwives. The nurse-midwife and the nurse-midwife coordinator: both work at a Public Health Centre on a daily basis. The nurse-midwife coordinator has additional responsibilities in administrative work, and she is the supervisor of all nurse-midwives in the Public Health Centre.

Practical experience has been divided into two categories; junior nurse-midwives with equal or less than ten years practical experience and senior nurse-midwives with more than ten years practical experience.

We divided nurse-midwives into three age categories: under 25 years old, between 25 until 50 years old, and over 50 years old. Working area is based on the district where the Public Health Centre is located. Nurse-midwife’s training during the last five years has been classified into three categories: training in management of anaemia in pregnancy; other kinds of training related to maternal health; and never been trained.

### Ethical consideration

Ethical approval was given by the Faculty of Medicine Universitas Gadjah Mada. Prior to the interview process, the interviewer explained the aim of the study to each participant and voluntarily participation in the study was confirmed. Written consent was obtained prior to all interviews. All participants had the right to withdraw their participation at any given moment.

## Results

Almost half of the 23 participating nurse-midwives were senior [Table 1]. All participating nurse-midwives were women. Eight of the 23 nurse-midwives worked as coordinators. 39% of the participants were between 25 – 50 years of age. Almost equal numbers of junior and senior nurse-midwives participated in the study. The nurse-midwives have been trained in various maternity service aspects during their initial training but none of them received any training concerning the management of pregnant women with anaemia.

**Table 1:** **Characteristics of participants**

**Characteristics**	**N** = **23**	%
**Nurse**-**midwife**’**s Position**		
Nurse-midwife coordinator	8	35
Nurse-midwife non coordinator	15	65
**Working Area**		
Gunungkidul district	5	21.7
Bantul district	5	21.7
Sleman district	4	17.4
Kota district	3	13.1
Kulonprogo district	6	26.1
**Age**		
<25 years	6	26.1
25 – 50 years	9	39.1
>50 years	8	34.8
**Practical experience**		
≤10 years	12	52
>10 years	11	48
**Training during last five years**		
Anaemia in pregnancy management	0	0
Other kinds of training related to maternal health	20	87
Never been trained	3	13

Data analysis identified three main themes among the experiences of the nurse-midwives: 1) the lack of competence and clinical skills; 2) cultural beliefs and low participation of family in the antenatal care programme; 3) insufficient facilities and support of staff in the Public Health Centres. Table 2 shows the data analytic framework of this study.

**Table 2:** **Data analytic framework**

**Theme Emerges**	**Topics**	**Classifications**	**Categories**	**Sub**-**categories**	**Code**
The lack of competences and clinical skills	Antenatal care standard implementation	Barriers	Too many procedures that have to be accomplished	Passed the procedure	Difficult to manage the time to accomplished all procedures
				Too many patients to be handled	Easy to feel irritable or impatient when having a long queing of patients that have to treated
			Unclear procedures guideline	Not sure to implement the procedure guideline	Hb test is not always be done to every women
				Afraid of making a mistake	Unclear when Hb test should be taken
					Doubt to take an action
			Preoccupied with administrative work	Writing some reports	A lot of papers works: writing many reports
		Facilitators	May delegate the task	Delegate the task to student	Asked the student to give health education
	Experience in anaemia prevention	Knowledge and skills competences	Unconfident	Not confident to give health education	Feel uncapable to deliver health information
				Not sure when detecting an early signs of anaemia	Doubtful with what has been done in order to detect an early signs of anaemia
					Doubts with iron tablet prescribing
Influence of cultural beliefs on family participation in antenatal care		Cultural competences	Difficult to manage	Food taboo for pregnant women	Meat, fish, or eggs is forbidden to be eaten
				Strong believes in traditional healer	Family took the woman to the traditional healer
				Pregnancy is female business	Unusual for man to participate in prenatal care
Insufficient facilities, resources and support of staff	Resources and facilities	Availabilty	Lack of learning resources	Learning resources is not available in PHC	Unavailable of booklets or other media as health information resources
			Staff shortages	Limited staff and facilities in PHC	Laboratory test can be delayed when the laboratory staff is out of duty
					PHC has one laboratory staff

### The lack of competences and clinical skills

The nurse-midwives mentioned their difficulties and expressed unease at providing health information to pregnant women. They worried that their explanation did not meet the patient’s need.

*"The most difficult thing I have done so far is to deliver health information to patients … I’m afraid that what I know is only a little bit … then … I could not answer patient’s questions …" (junior nurse-midwife, 22 years old)*

Besides communication skills, all nurse-midwives felt that patience, empathy, and politeness were important. The more experienced nurse-midwives said that their patience often decreased or was tested when they met a pregnant woman who did not understand or would not listen to what they tried to explain to her. These senior nurse-midwives said that they therefore readily delegated this task to a student.

*“I frequently feel irritable when the patient does not understand what I’m saying … Whether I don’t give the information clearly … whether the patient is uneducated … So, I ask the student to teach the patient …if not … let the nutritionist do so [give health information to the woman] …”(senior nurse-midwife, 53 years old, coordinator)*

Some of the nurse-midwives expressed doubts about their clinical skills to detect early signs of anaemia in pregnancy. They also mentioned that they still need a lot of practice to perform accurate investigations to detect anaemia early in pregnancy.

*“....I feel that the knowledge I got from college is not enough, sometimes…I’m not sure to what I have done…detecting early signs of anaemia is not as simple as I learnt at college…”(junior nurse-midwife, 23 years old)*

### Influence of cultural beliefs on family participation in antenatal care

The nurse-midwives were confronted with the strength of cultural beliefs concerning food taboos such as pregnant women are forbidden to eat meat, fish or eggs and the family’s attitudes toward pregnancy such as pregnant women should take care of herself and her pregnancy, but the husband will take the necessary decisions regarding to his wife’s pregnancy. For example, the husband will decide where the pregnant woman should attend her antenatal care (to the health professional or to the traditional healer) and where the pregnant woman should give birthing process (at home or at the public health centre). According to the nurse-midwives, the strength of cultural believes among pregnant women and her family members result in an unhealthy life style of pregnant women. In addition, they reported a lack of health information resources such as booklet, leaflets, or health education that can be used by the women, husbands and family members to improve their knowledge about anaemia in pregnancy.

*“It is rare that the patient is accompanied by the husband … if he is there … he will not join in the antenatal care room … but he waits in the parking area … he thinks that it is a women’s business …”(senior nurse-midwife, 40 years old)*

*“… A difficult one is when the woman and her family have strong believes on dukun (traditional healer) … they (family members including husband) do not want to report the pregnant woman’s health problem to us but will go to the dukun....”(senior nurse-midwife, 54 years old, coordinator)*

*“… I have to explain many times to the pregnant women that it is only a myth … many pregnant women do not want to consume meat or fish (they do not want to consume because it is forbidden or food taboo for pregnant woman) … because they believe that it will make odour in their blood …”(senior nurse-midwife, 47 years old, coordinator)*

The majority of the nurse-midwives believed the husband and family members had an important influence on pregnant women’s lifestyle. For example they thought husband and family remind a woman to take her daily iron tablets, and encourage her to make regular visits to the nurse-midwife. Nurse-midwives felt it was important that pregnant women were accompanied to their antenatal check-ups by family members so that they can be encouraged to participate in her care.

*For me … it is better if the husband can join in [in the antenatal room] … to listen when I’m doing antenatal care … and I can ask him to remind his wife to take the tablet [iron tablet] … sometimes she forgot … or she doesn’t want to take the pills because it can induce nausea … (senior nurse-midwife, 45 years old)*

One nurse-midwife said that involving a husband or family members will give her an additional task in antenatal care, because she has to spend extra time to answer the husband’s questions.

*“… I do not believe that a husband involved in antenatal care will be helpful … Based on my experiences … is contrary … a husband in the antenatal room makes my work doubled … yes … because usually men are asking more than women …”(senior nurse-midwife, 53 years old)*

However, most nurse-midwives believed that they can overcome inappropriate cultural beliefs by providing health information and actively involving the husbands and/or family member in antenatal care.

### Insufficient facilities, resources and support of staff

All nurse-midwives mentioned that although equipment such as height and weight scales, portable ultrasound equipment, and stethoscopes were available at the Public Health Centre, they felt they were of insufficient quantity and quality. Some Public Health Centres have ultrasound equipment available, but the nurse-midwives said they have not been trained to use it.

Furthermore they expressed they were hindered in their work because of insufficient facilities and staff. They mentioned that blood and urine testing were available, but only one person in every Public Health Centre is capable to handle laboratory tests. As a consequence, the nurse midwives felt doubtful about being able to implement an adequate standard of antenatal care.

All nurse-midwives mentioned that they prescribed iron tablets as a routine procedure to all pregnant women without exception. But they mentioned that the technical procedure did not give any clarity about timing and indications of Hb testing.

*It depends .... there are some that have the Hb measurement and some that don’t .... it is not clear when they should be measured (Hb) .... because it’s not written in the technical procedure… (Nurse-midwife, 37 years old)*

All nurse-midwives and nurse-midwives coordinators mentioned that the large workload and insufficient staff numbers prevent them from carrying out their work according to procedures.

*“What is a bother is having to do a lot of writing in this format, not only writing the daily reports, but it’s even more of a bother if there is a request from the departments for data for the annual report…”(senior nurse-midwife, 55 years old, coordinator)*

For example they understand that checking Hb is one of the components of standard antenatal care, but they reported that their compliance with the standard varied, depending on their workload.

*“… sometimes I did not check a patient’s condition in detail … and I forgot to check the woman’s Hb, mostly when so many patients are queuing outside …”(senior nurse-midwife, 47 years old, coordinator)*

## Discussion

### Main finding

According to the perceptions of nurse-midwives there are three factors which hinder the adequate prevention of anaemia in pregnancy in Public Health Centres: the substandard antenatal care; the competences to cope with cultural beliefs; and the need of a more comprehensive approach to antenatal care.

### Substandard antenatal care

The nurse-midwives perceived that insufficient facilities, high work load, lack of training opportunities and learning resources for the nurse-midwives, and limited supporting staff appear to be the most important barriers for better antenatal care services in Public Health Centres. Different perception arised from pregnant women, they perceived that the substandard antenatal care they have recieved in Public Health Centres is related to the services free of charges [19].

Other studies in South Sumatra, North Jakarta, Kebumen, and Central Java highlight similar factors which affect the success of maternal health care programmes. In these studies, ineffectiveness of nurse-midwives’ work placement coupled with the lack of training opportunities, and the lack of learning resources, resulted in substandard care for pregnant women [22,23]. In West Java, only 18% of nurse-midwives in Public Health Centres have been trained in early risk detection in pregnancy and normal delivery care and 4% on live saving skills [24]. Other studies mentioned that 90% of nurse-midwives in Indonesia have not had any opportunities for continuing education [25]. Moreover, the Ministry of Health stated that the skills of 60% the nurse-midwives’ were misused and underused because of the absence of a clear job description [26]. Therefore, the competence of nurse-midwives in taking care for pregnant women with anaemia can not be guaranteed.

### Coping with cultural beliefs

Nurse-midwives seem to struggle with how to cope with the women’s and their families’ cultural beliefs. The strength of cultural beliefs enormously influences women’s healthy lifestyle and family participation in antenatal care programme [27]. Cultural beliefs on food taboo for pregnant women contributes to the incidence of anaemia in many countries [28-32]. The strength of cultural beliefs enormously influences women’s healthy lifestyle and family participation in antenatal care programme [27]. Therefore the nurse-midwives must acquire the appropriate knowledge and skills in cultural sensitive care [33].

To gain more insight into the level of health literacy of pregnant women and their families, nurse-midwives need to explore women’s knowledge of what constitutes a healthy lifestyle as well as their cultural beliefs [34]. Communication skills are essential to bridge the cultural diversity between health care providers and their clients [35,36]. Communication skills have been emphasized as one of nurse-midwives’ core competencies [37,38]. However, our study reveals that the majority of nurse-midwives express a need for more training in communication skills. They would like to feel more confident by being competent in delivering health information about anaemia and a healthy life style to pregnant women and being able to bridge the cultural beliefs. Moreover they like to be more competent in an early detection of anaemia in pregnancy.

Our study results are supported by other studies which concluded that nurse-midwives’ communication skills and the interaction with the client need to be improved and highlighted that basic medical skill also important to be improved [24,25,34,39].

### A more comprehensive approach to antenatal care

According to the nurse-midwives, in some cases the women and families prefer to visit a traditional healer rather than to visit the nurse-midwives for antenatal check ups. The reasons of choosing traditional healer because they do not have to pay more to the traditional healer, easy to be accessed, the myth of traditional healer, and they will be helped for household chores [40,41]. Health workers’ attitudes, delay in providing care, substandard care, and unavailability of skilled attendant are some factors that rises unsatisfaction to the antenatal care services [42].

Anaemia in pregnancy needs immediate attention by combining some strategies that can comprehensively combat the disease [43]. A combination of strategies could include the womens’ health knowledge improvement, husbands or family members participation in antenatal care programme, positive beliefs and practices stimulation, professional attitudes and adequate antenatal treatment [31,42,44-46].

### Limitations and strengths

Our study presents data on those who carry out family health policy relating to the prevention of anaemia during pregnancy at Public Health Centre level. Due to the qualitative nature of this study, the results represent the situation in Yogyakarta Special Province. However we did succeed in including a group of nurse midwives who represent a broad representation of the Public Health Centres in the Districts, from junior to senior level, from non-coordinator to coordinator. Therefore we assume that our findings are applicable to health care systems with similar conditions in other regions and countries as well.

## Conclusions

The management of anaemia during pregnancy in Public Health Centres in Yogyakarta Special Province is constrained by three factors. First factor is the nurse-midwives’ competencies in communication and clinical skills to manage pregnant women with anaemia. The second is the husband and family involvement in antenatal care was constrained by the strength of cultural beliefs and lack of health information and the last is unfavourable work environment of the Public Health Centres made it difficult to apply antenatal care the pregnant womens’ need.

The success of a maternal health care programme not only depends on the nurse-midwives skills, but should also be supported by the organisation where the nurse-midwives work. A healthy and supportive organisation knows its employees, understands their needs and maintains and improves their level of competence by providing a combination of facilities, learning resources and training for their employees [47].

Based on our research findings, we conclude that pregnant women with anaemia need to be cared for by using a more comprehensive approach which can empower nurse-midwives and pregnant women in order to improve maternal and child well being.
